# Origin of Polyploidy: Results of Laboratory Crosses Between Green Toad (*Bufotes*) Diploid Species

**DOI:** 10.3390/ani16193082

**Published:** 2026-09-30

**Authors:** Kseniya A. Matushkina, Anton O. Svinin, Dmitriy V. Skorinov, Nikolai D. Aksenov, Artem A. Kidov, Spartak N. Litvinchuk

**Affiliations:** 1 Timiryazev Agricultural Academy, Russian State Agrarian University, 49 Timiryazevskaya Street, Moscow 127550, Russia; matushkinaka@gmail.com (K.A.M.); kidov_a@mail.ru (A.A.K.); 2 Institute of Cytology, Russian Academy of Sciences, Saint-Petersburg 194064, Russia; skorinovd@yandex.ru (D.V.S.); aksenovn@gmail.com (N.D.A.); litvinchukspartak@yandex.ru (S.N.L.)

**Keywords:** allopolyploidy, Anura, Bufonidae, hybridization, reticulate evolution

## Abstract

Unreduced gametes contribute to the emergence of polyploid plant and animal species. Interspecific diploid and polyploid hybrids can utilize clonal or hemiclonal reproduction methods, in which one or all parental genomes are transmitted to offspring without recombination. To elucidate mechanisms supporting the coexistence of parental species and polyploid hybrids, we conducted artificial crossing experiments between two diploid species of green toads. We determined the ploidy levels of all hybrids resulting from the crosses. The offspring included di-, tri-, tetra-, penta-, and hexaploid individuals. In the first generation, all hybrids were generally diploid. In the second generation, many polyploid individuals appeared in the offspring. Hybrids (even in a single cross) produced tadpoles with different ploidy levels due to gametes with different genomic compositions. Males of triploid hybrids produced predominantly haploid gametes, while females produced diploid ones. We propose a model for the origin of polyploid species in the genus *Bufotes*.

## Simple Summary

The formation of unreduced gametes is a key mechanism of polyploidization in both plants and animals. Such gametes can give rise to polyploid species. Polyploid individuals, in turn, can produce unreduced gametes, facilitating the transition to higher ploidy levels. In interspecific diploid and polyploid hybrids, clonal (or hemiclonal) reproduction allows the transmission of parental genomes to offspring unchanged without recombination. To understand how parental species and their polyploid hybrids can coexist, we conducted controlled crosses between two diploid species of green toads. We determined the ploidy of all offspring. The progeny included di-, tri-, tetra-, penta-, and hexaploid individuals. While first-generation hybrids were predominantly diploid, the second generation contained numerous polyploids. Importantly, males and females from the same cross produced gametes with different genome compositions, resulting in offspring with heterogeneous karyotypes. Triploid males mainly produced haploid gametes, whereas triploid females produced diploid gametes. Based on our results, we propose a model of polyploid speciation in the genus *Bufotes* that emphasizes the role of gametic variation and hybridization in shaping genomic diversity and stimulating evolutionary innovation.

## 1. Introduction

Polyploidy plays an important role in the evolution of many groups of organisms [1]. Polyploids can be divided into two categories: allopolyploids, which are hybrids, and autopolyploids, which are individuals that have more than two sets of chromosomes of the same species [2,3]. Mechanisms of polyploid speciation are an important area of research in evolutionary biology [4,5,6]. Polyploidy and hybridization have profound effects on genome structure and gene regulatory networks [7,8,9,10,11,12]. The evolutionary history of several vertebrate groups includes numerous episodes of whole genome duplication [13,14,15]. Although polyploidy is frequently documented across various vertebrates, particularly in fishes and reptiles [16,17,18,19,20,21,22], it is also widely distributed among amphibians [23,24,25,26,27]. The widespread occurrence of polyploidy in this group can be explained by their considerable genome plasticity, diversity of sex-determination systems, species-specific reproductive traits, and some other biological factors [28,29,30].

In amphibians, polyploidy frequently arises through hybridization events [14,31,32,33,34,35,36,37,38,39,40,41,42]. However, the detailed molecular and cellular mechanisms involved in this process remain poorly understood [42]. A proposed model for this mode of hybridogenetic (or reticulate) speciation suggests that in its initial stages, diploid and triploid hybrids can reproduce clonally (i.e., parental genomes are transmitted to offspring without recombination). In the final stages of this speciation, tetraploid species that reproduce sexually arise [14,43].

Among anurans, polyploid speciation is particularly common in the genus *Bufotes* Rafinesque, 1815, inhabiting almost the entire Western Palearctic [44]. The genus consists of 7–10 diploid and 6–8 polyploid species [45,46,47,48,49]. Diploid species of the genus can be divided into two groups between which the divergence time is about 16.5 million years [46]. The first group consists of 4–7 species of the subgenus *Bufotes*, and the second of three species of subgenus *Calliopersa* Safaei-Mahroo et Ghaffari, 2020 [49,50,51]. All polyploid species are bisexual and are represented by 3–4 triploid and 3–4 tetraploid species [46,48], which are hybrids between three species of the subgenus *Bufotes* on one side (the maternal species) and *B*. *latastii* (Boulenger, 1882) from the subgenus *Calliopersa* on the other side (the paternal species). Triploid species always have two genomes of the paternal species and only one of the maternal species [46]. They reproduce hemiclonally, transmitting to their offspring only one paternal genome in haploid sperm and both parental genomes in diploid oocytes [52,53]. Tetraploids are bisexual, and both sexes produce diploid gametes with both parental genomes [45,46,54].

A central challenge in understanding polyploid speciation lies in elucidating the specific mechanisms and conditions that enable the emergence of new polyploid lineages [30,44,46]. The genus *Bufotes*, which includes a complex of diploid and polyploid species, provides a compelling model system to investigate this process [46,54]. To experimentally study hybridogenesis and reticulate speciation within the genus, we focused on two diploid species: *B*. *sitibundus* (Pallas, 1771) from the subgenus *Bufotes* and *B*. *latastii* from the subgenus *Calliopersa*. The first species inhabits a wide range spanning the Middle East, the Anatolian, Arabian, and Sinai peninsulas, some Aegean islands, the Caucasus, northern and western Kazakhstan, the southern Urals (with adjacent Siberia), and the Volga River drainage of European Russia, and is sometimes classified as a subspecies of *B*. *viridis* (Laurenti, 1768) [47,55]. In contrast, *B*. *latastii* is an endemic species found only in the Indian and Pakistani Himalaya [56]. It is assumed that these two diploid species participated in the formation of such triploid species as *B*. *zugmayeri* (Eiselt and Schmidtler, 1973) and *B. pseudoraddei* (Mertens, 1971), as well as such tetraploid species as *B*. *oblongus* (Nikolskii, 1896) and possibly *B*. *zamdaensis* (Fei, Ye et Huang, 1999) [46]. For this purpose, various types of laboratory hybrids were obtained between these two species in order to study ploidy, survival, various developmental parameters, fertility, and sex ratio in their offspring. These data contribute to a better understanding of reticulate speciation and processes underlying the induction of polyploidy in this group of amphibians.

## 2. Materials and Methods

Several laboratory generations of *B*. *latastii* (Tangmarg, India; 34.049° N, 74.428° E) and *B*. *sitibundus* (Shamakhi, Azerbaijan; 40.897° N, 48.364° E) have been kept in the Laboratory of Zooculture of the Department of Zoology of the Russian State Agrarian University—Moscow Agricultural Academy—named after M. V. Timiryazev (Moscow, Russia) since 2012 [57,58]. Crosses between these two species were carried out from 2013 to 2024. We used 2–4-year-old individuals for breeding experiments. In total, we used 17 females and 15 males of *B*. *latastii*, as well as 8 females and 11 males of *B*. *sitibundus* in 85 laboratory crossings. We performed all manipulations with toads in accordance with national and international guidelines. The study’s compliance with international ethical standards was confirmed by the Bioethics Commission of the Russian State Agrarian University—Moscow Timiryazev Agricultural Academy (Protocol No. 1, dated 6 September 2019).

In the laboratory, we kept toads in plastic containers (40 × 20 × 25 cm) at room temperature. We used wet viscose napkins (FineLife, Abrasive Technology LLC, Sosnovy Bor, Russia) as a substrate. To regulate the light regime, we used fluorescent lamps with an ultraviolet spectrum (14 h a day, 30 W, 1150 lm; ReptiLight, NARVA, Brand-Erbisdorf, Germany). During the entire period of the experiment, we used the two-spotted cricket, *Gryllus bimaculatus* (De Geer, 1773), the yellow flour beetle, *Tenebrio molitor* (Linnaeus, 1758), the large wax moth, *Galleria mellonella* (Linnaeus, 1758), the speckled cockroach, *Nauphoeta cinerea* (Olivier, 1789), and the Turkestan cockroach, *Blatta lateralis* (Walker, 1789), grown in the laboratory, as a food for adult toads. Before feeding the toads, we dusted insects with a mineral premix (Micro Calcium, JBL GmbH&Co., Neuhofen, Germany). Young toads were fed every day and adult toads only 3–4 times a week.

In accordance with seasonal cycles, the animals hibernated annually. After two weeks of fasting, we placed toads in plastic containers with a 10 cm layer of moistened oak leaves, and then the temperature was gradually reduced. The duration of hibernation ranged from 30 to 119 days. The air temperature during this period fluctuated from 0.3 to 16 °C (average 10.2 ± 0.1 °C; SD = 2.6). At the end of wintering, the temperature was gradually raised to 20 °C.

Then, we placed toads in pairs in plastic containers (39 × 28 × 28 cm) with a water level of 10 cm to spawn. Foam sponges were used as imitation of islands. The spawning of toads took place at room temperature. To speed up and synchronize their reproduction, we injected each toad with a solution of surfagon (Mosagrogen, Moscow, Russia; a synthetic equivalent of the hypothalamic neurohormone GnRH) into an axillary region. Injections were repeated up to 5 times in 24 h increments until successful spawning. The dose of the hormone in each injection was 12.5 µg.

The embryonic and larval development took place at the temperature of 15–20 °C (average 18.0 ± 0.1 °C; SD = 2.6). We changed water in containers with eggs, embryos, and pre-larvae (before they started feeding) every other day (1/3 of the container’s volume) and with tadpoles daily (2/3 of the container’s volume). The main food for tadpoles was aquarium fish food (Marine Flakes, Tetra GmbH, Melle, Germany) and leaf spinach (Vitamin, Miratorg, Domodedovo, Russia) [58]. The duration of embryonic and larval developments was calculated using mean values for each breeding experiment. The duration of embryonic development was considered from the laying by the female of the egg string until the start of active feeding of most tadpoles, and the duration of larval development was from the beginning of active feeding of most tadpoles until the completion of metamorphosis.

For unhindered access to land, we placed metamorphosed toads after forelimb appearance to inclined plastic containers with a water level of 3–4 cm in the deepest part. Two or three days after complete resorption of the tail, we put the young toads into dry plastic containers with wet viscose napkins as a substrate. The food for the young animals was the larvae of the two-spotted cricket and the Turkestan cockroach of suitable sizes. Due to the uneven growth of juveniles, we had to regularly sort the toads to avoid cannibalism and oppression of small individuals.

All meristic characters (length of pre-larvae (stages 18–20 of [59]), larvae (stage 25), and metamorphosed toads, thickness of egg strings, and egg diameter) were measured with a digital caliper with an error of 0.1 mm. The mass of adult animals and metamorphs was determined on scales with an error of 0.01 g. The egg number in clutches was determined by a total count. We tested for normality of the distribution using the Kolmogorov–Smirnov test with the Lilliefors correction. We performed a one-way analysis of variance (ANOVA), followed by the Scheffe post hoc test for pairwise comparisons. The analyses were conducted using Statistica 8.0 and R packages ggplot2 (v4.0.3), readxl (v1.5.0.1), and dplyr (v1.2.1) in RStudio 1.3.1093.

For reliable determination of ploidy of laboratory hybrids, the nuclear DNA content (=genome size) in blood cells of 292 individuals was examined by DNA flow cytometry (Table 1). Among parent species, 25 individuals of *B*. *latastii* and 4 of *B*. *sitibundus* were studied (Table 2). Additionally, to study the composition of sperm cells, we analyzed, by DNA flow cytometry and microscopic examination of the suspension, three males of the F2 hybrid between a *B*. *latastii* female and a *B*. *sitibundus* male and five males of the F2 hybrid between *B*. *sitibundus* females and *B*. *latastii* males. To minimize suffering, individuals were euthanized by a 1% solution of 3-aminobenzoic acid ethyl ester (MS 222). We took blood samples immediately from the heart and stored them before experiments in Versene solution (phosphate buffer containing 0.7 mM EDTA, pH = 7.3–7.7; Biolot, St. Petersburg, Russia) at the temperature of ~5 °C.

DNA flow cytometry was conducted using a CytoFLEX flow cytometer, Beckman Coulter, Inc., Brea, CA, USA (EB), and/or a laboratory model of a flow cytometer (EB + OM) developed at the Institute of Cytology, Russian Academy of Sciences (St. Petersburg, Russia; https://patents.google.com/patent/SU1056008A1/ru; accessed on 1 June 2026), based on a Lumam-II fluorescence microscope (Lomo, St. Petersburg, Russia) with a mercury lamp as a light source. The genome size in relative units was determined for each individual as the ratio of the average value of the peak of the studied cells on the DNA histogram to the peak for the reference standards. To analyze measurement results, the CytExpert 2.0 (Beckman Coulter) and BARS 1.0 (Institute of Cytology, Russian Academy of Sciences) programs were used. Peripheral blood cells of the grass frog (*Rana temporaria* Linnaeus, 1758), collected in Leningradskaya Oblast’, Russia (average genome size is 10.32 pg by [60]), and male house mouse (*Mus musculus* Linnaeus, 1758) splinocytes (laboratory line C57B1; 6.80 pg by [61]) were used as reference standards. Tested cells were mixed with reference cells and assayed simultaneously. Therefore, in such a mixture, both kinds of cell samples were stained and measured in the same conditions.

The total concentration of cells in the suspension was approximately 106 cells/mL. To 500 μL of the reference standard cell mixture and 500 μL of the test species cell mixture, 5.5 μL of a 0.3 M aqueous solution of MgCl_2_, 5.5 μL of 1% aqueous Triton X-100 solution, and 5.5 μL of 1% aqueous ethidium bromide (EB; for measurement on CytoFLEX), or a mixture of 2.5 μL of 1% aqueous olivomycin solution and 5.0 μL of 1% aqueous solution of ethidium bromide (EB + OM; for measurement on a laboratory model of a flow cytometer) were added successively. Because of the GC specificity of olivomycin, the genome size estimates obtained by staining nuclei with a mixture of this dye and ethidium bromide were overestimated. Therefore, we multiplied them by an experimentally calculated coefficient, which on average for this genus amounted to 0.960. The standard staining duration ranged from 10 to 60 min at room temperature (~21 °C). Details of the method were previously published [60,62].

For karyological examination, five individuals of *B*. *sitibundus* (7 plates), seven *B*. *latastii* (12), and 16 laboratory hybrids (53 plates for 4 individuals of *B*. *sitibundus* ♀ × *B*. *latastii* ♂, 6 F2 (*B*. *sitibundus* ♀ × *B*. *latastii* ♂), 1 F3 (*B*. *sitibundus* ♀ × *B*. *latastii* ♂), 2 B1 (*B*. *sitibundus* ♀ × *B*. *latastii* ♂) ♀ × *B*. *latastii* ♂, and 4 B1 *B*. *latastii* ♀ × (*B*. *sitibundus* ♀ × *B*. *latastii* ♂) ♂) were injected with 0.3–0.5 mL of a 0.4% aqueous solution of colchicine one day before tissue sampling. Before fixation, intestine cell samples were kept in hypotonic 0.07 M KCl solution for 12 min. Then, tubes with tissue samples were centrifuged at 755 g in hypotonical solution for 2 min, supernatant liquid was poured out, and samples were fixed. Fixative was changed three times each 30 min of incubation. Later, treated tissue fragments were kept in 60% acetic acid water solution, and cell suspension was dropped on glass slides heated up to 60 °C. Slides were stained using Giemsa, Ag-NOR-, DAPI/distamycin A-, and Q-staining methods [63].

## 3. Results

### 3.1. Genome Size Variation

**Figure 1:**
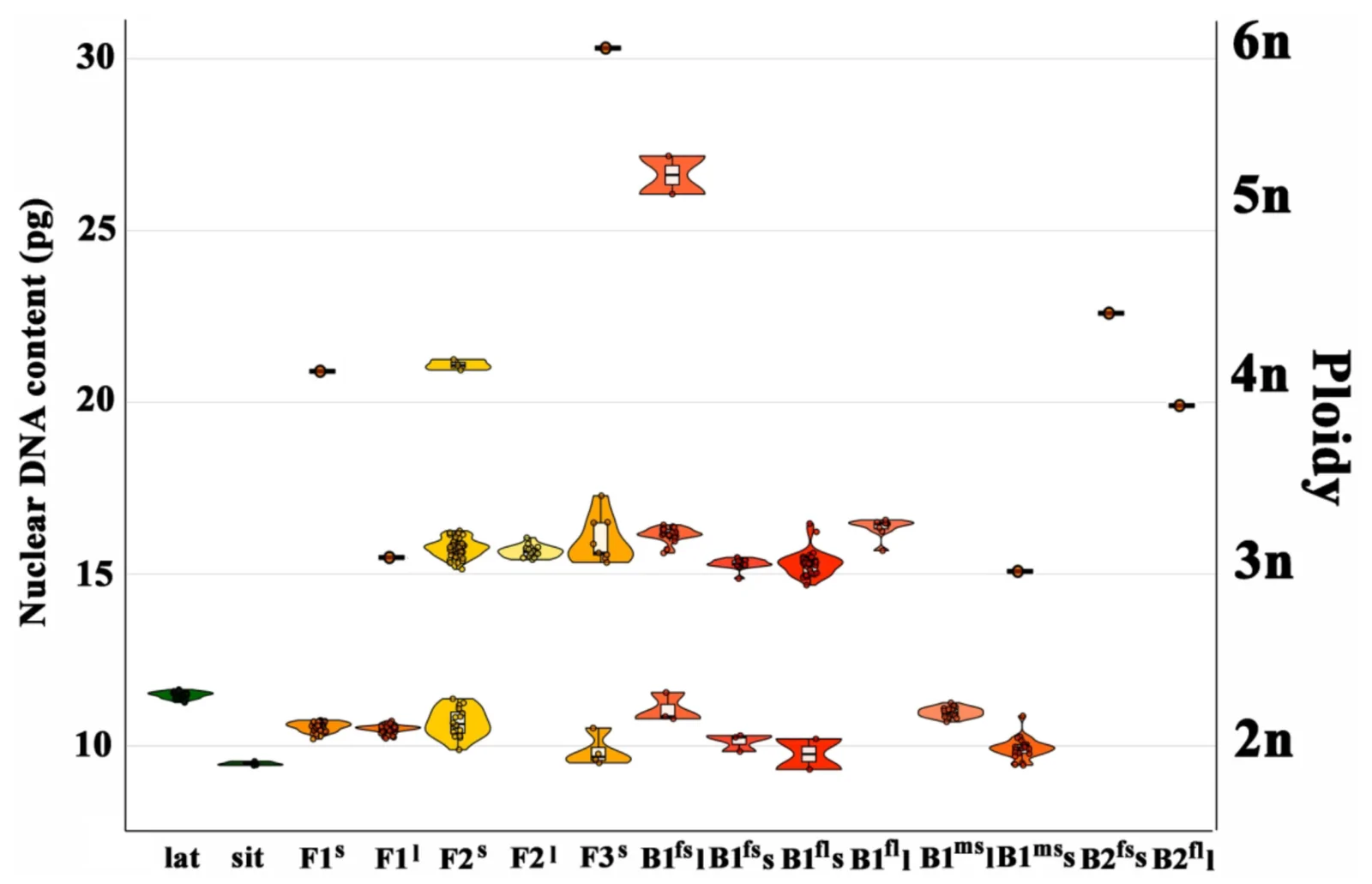
The nuclear DNA content variation (violin plots include boxplots with mean values and interquartile ranges) in *Bufotes latastii* (lat), *B. sitibundus* (sit), and their hybrid offspring across various (2–6*n*) ploidy levels. The color scheme represents different hybrid types (parental species are shown in green, F1–3 hybrids are orange–yellow, and B1–2 backcrosses are coral–red). Abbreviations for various types of hybrids are given as in Table 1.

The average nuclear DNA content of *B*. *latastii* (11.3–11.6 pg) was 19% greater than that of *B*. *sitibundus* (9.4–9.5 pg), and their variability did not overlap (Table 2; Figure 1). In diploid hybrids the nuclear DNA content was between that of the parent species and varied from 9.3 to 11.6 pg. In polyploid offspring, the genome size gradually increases from triploids (14.7–17.3 pg), tetraploids (19.9–22.6 pg), and pentaploids (26.0–27.1 pg) to a hexaploid (30.2 pg) without overlapping, which allows them to be classified according to each of the ploidy levels (Figure 1). Within these levels, there is also variability, which mainly depends on the contribution of genomes of each of the parental species. As a rule, genomes with a greater contribution from *B*. *latastii* have higher nuclear DNA content than genomes dominated by *B*. *sitibundus* (Table 2).

We analyzed the genome size of a suspension obtained from testes of three triploid F2 hybrid males, obtained by crossing a *B. latastii* female and a *B. sitibundus* male. Haploid spermatozoa (6% of the total number of cells analyzed in the suspension) were detected in only one male. All other cells (94%) were triploid. Microscopic examination of the suspension of this male revealed the presence of normal spermatozoa in the smear. No sperm were detected in the other two males. All males had two normal testes.

**Figure 2:**
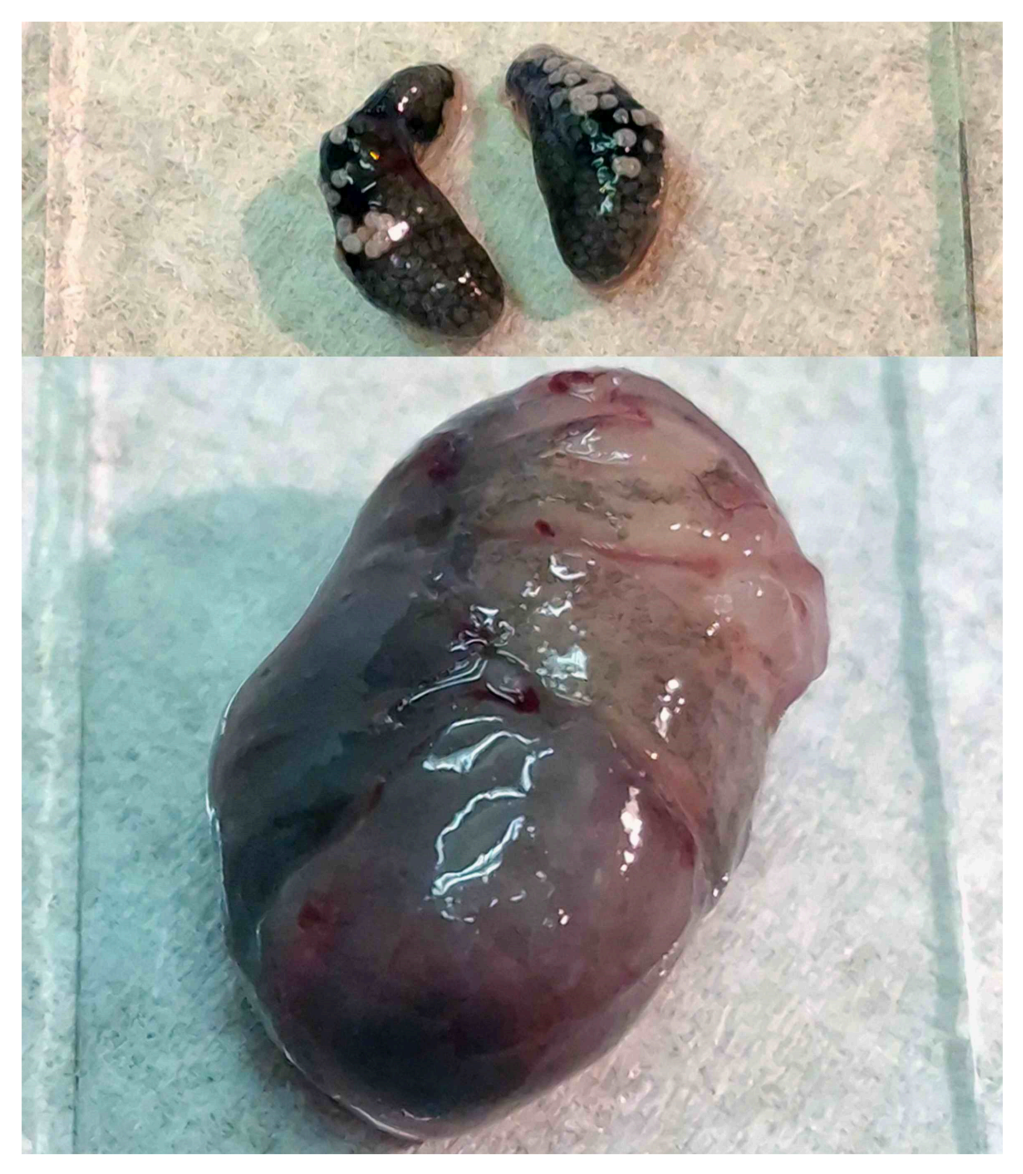
The top image shows two normal testes of a triploid F2 hybrid male obtained by crossing *B. latastii* females and *B. sitibundus* males and the bottom image shows one abnormal testis of a diploid F2 hybrid male obtained by crossing *B. sitibundus* females and *B. latastii* males. Both photographs were taken at the same scale (the same 75 mm-wide glass slide under the testes).

We also examined the genome size in suspensions obtained from the testes of one diploid and four triploid F2 hybrid males obtained by crossing *B. sitibundus* females and *B. latastii* males. In a diploid male we found only diploid cells, and in triploid males we found only triploid cells. Microscopic examination of these suspensions revealed no sperm. The triploid males had two normal testes, while the diploid male had only one abnormal testis, which was significantly larger than normal (Figure 2).

### 3.2. Structure of Karyotypes

**Figure 3:**
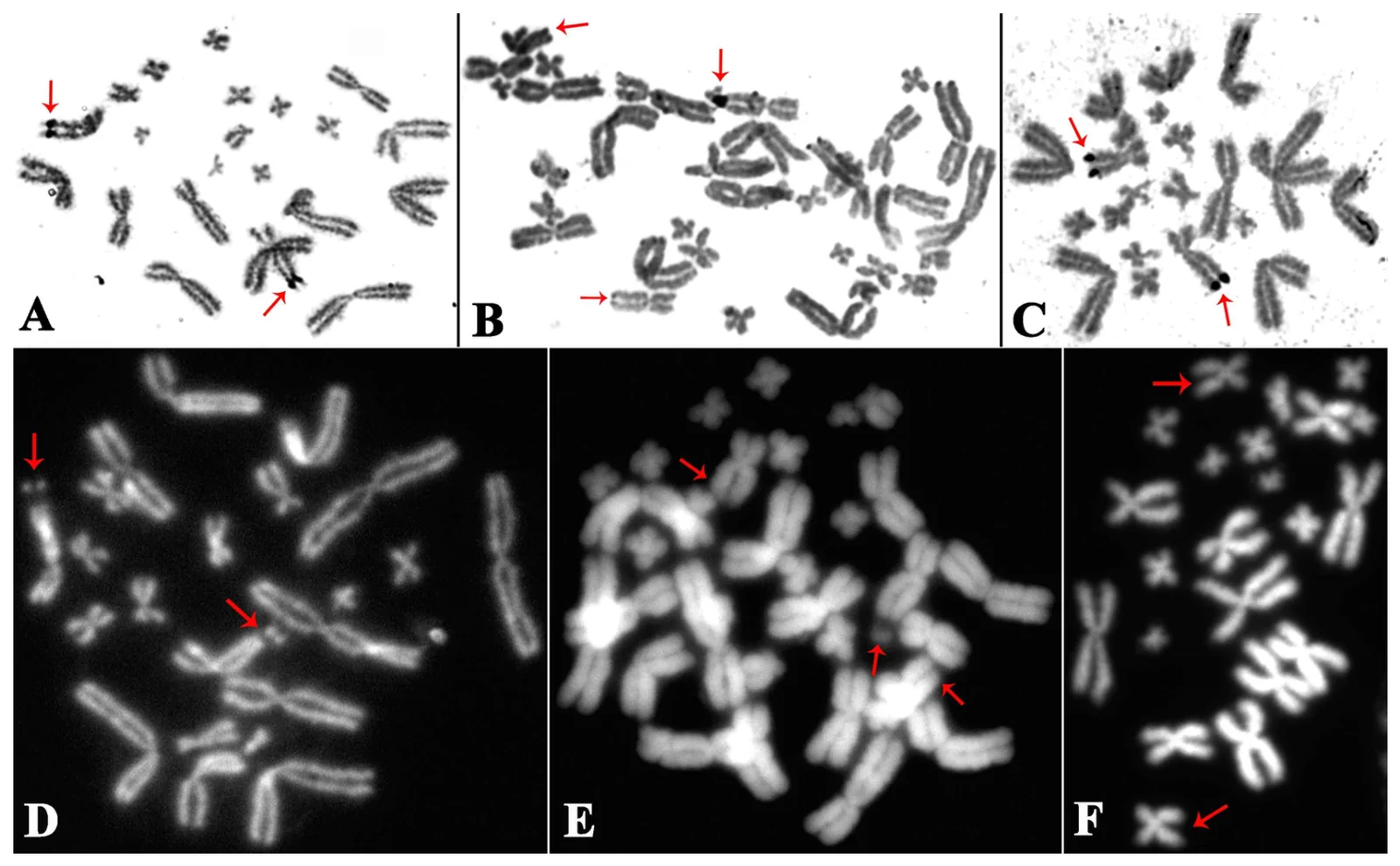
Ag-NOR- (**A**–**C**) and DAPI/distamycin A-stainings (**D**–**F**) of chromosomes of *Bufotes latastii* (**A**,**D**), *B. sitibundus* (**C**,**F**), and their triploid hybrids ((**B**,**E**) are two individuals of the F2 hybrid of *B. sitibundus* ♀ × *B. latastii* ♂). Red arrows indicate the location of NORs.

The diploid karyotypes of *B. latastii* and *B. sitibundus* consist of 22 biarmed chromosomes (2*n* = 22; NF = 44; Figure 3). Six chromosome pairs were large (pairs 1–6; relative length > 10.0%) and five were small (pairs 7–11; <5.5%). In all studied individuals of *B. latastii*, the 4th, 5th, and 7th pairs of chromosomes were submetacentric. In *B*. *sitibundus*, only the 4th pair of chromosomes was submetacentric. All other chromosomes were metacentric. The average chromosomal formulae were 2*n* = 16m + 6sm = 22 in *B. latastii* and 2*n* = 20m + 2sm = 22 in *B*. *sitibundus*. The positively Ag-NOR-stained regions were revealed on the long arm of chromosome 5 in the subterminal region in *B. latastii* and in the terminal region on the long arm of chromosome 6 in *B. sitibundus* (Figure 3). In *B. latastii*, DAPI/distamycin A-staining revealed positive bands on the short arms in the pericentromeric region on the 5th (NOR-bearing) pair of chromosomes, in the medial region of short arms on the 7th and 9th pairs, and in the submedial region of the long arms 8th pairs. Also, positive bands can sometimes be found in the submedial parts of the short and long arms of the 10th and on the long arms of the 11th pairs. In *B. sitibundus*, we found positive bands only in the pericentromeric region of the long arms of the first pair of chromosomes. DAPI/distamycin A- and Q-stainings revealed a shaded region in the subterminal region of the long arms of the 5th pair of chromosomes for *B. latastii* that stained with Ag-NOR (Figure 3). However, in *B. sitibundus* this shaded region is usually not visible, as it is located in the terminal region of the NOR-bearing chromosome.

Our data on the ploidy of laboratory hybrids, based on karyological characteristics and genome size, were completely consistent. Among the first-generation hybrids *B. sitibundus* ♀ × *B. latastii* ♂, three individuals were diploid (2*n* = 22) and one was triploid (3*n* = 33). In two diploid and one triploid individuals, on one chromosome only NOR was detected on the long arm of chromosome 5 in the subterminal region (like in *B. latastii*). And only in one diploid individual were two NORs found: in the subterminal (as in *B. latastii*) part of the chromosome bearing NOR, and in the terminal (as in *B. sitibundus*) region of the other.

Among the second-generation hybrids *B. sitibundus* ♀ × *B. latastii* ♂, five individuals were triploid (3*n* = 33) and one was tetraploid (4*n* = 44). In triploid individuals, only on one chromosome was NOR detected on the long arm of chromosome 5 in the subterminal region (like in *B. latastii*; Figure 3). In the tetraploid individual, we detected NORs in two chromosomes on the long arm of chromosome 5 in the subterminal region (like in *B. latastii*). DAPI/distamycin A-staining of triploids revealed positive bands on only two of three chromosomes on pairs 7–9 and 11 (like in *B. latastii*).

In a diploid hybrid of the third-generation *B. sitibundus* ♀ × *B. latastii* ♂, the nucleolar organizer (Ag-staining) was found only in the subterminal region of one of the two chromosomes 5 (like in *B. latastii*).

Two F1 backcrosses (*B. sitibundus* ♀ × *B. latastii* ♂) ♀ × *B. latastii* ♂ studied by us were triploid. We detected NOR (Ag-staining) on only one of the three NOR-bearing chromosomes (like in *B. latastii*). F1 backcrosses *B. latastii* ♀ × (*B. sitibundus* ♀ × *B. latastii* ♂) ♂ were diploid. DAPI/distamycin A- and Q-staining revealed a shadowed region in the subterminal part of the long arm of one of the two NOR-bearing homologs, at the same position as in *B. latastii*. DAPI-positive bands were detected on both chromosomes 7 and 9, but only on one of the two homologous chromosomes 8 and 11.

### 3.3. Results of Crossings

#### 3.3.1. Hybrids Between *B. sitibundus* Females and *B. latastii* Males

##### Hybrids of the First Generation (F1^s^)

**Figure 4:**
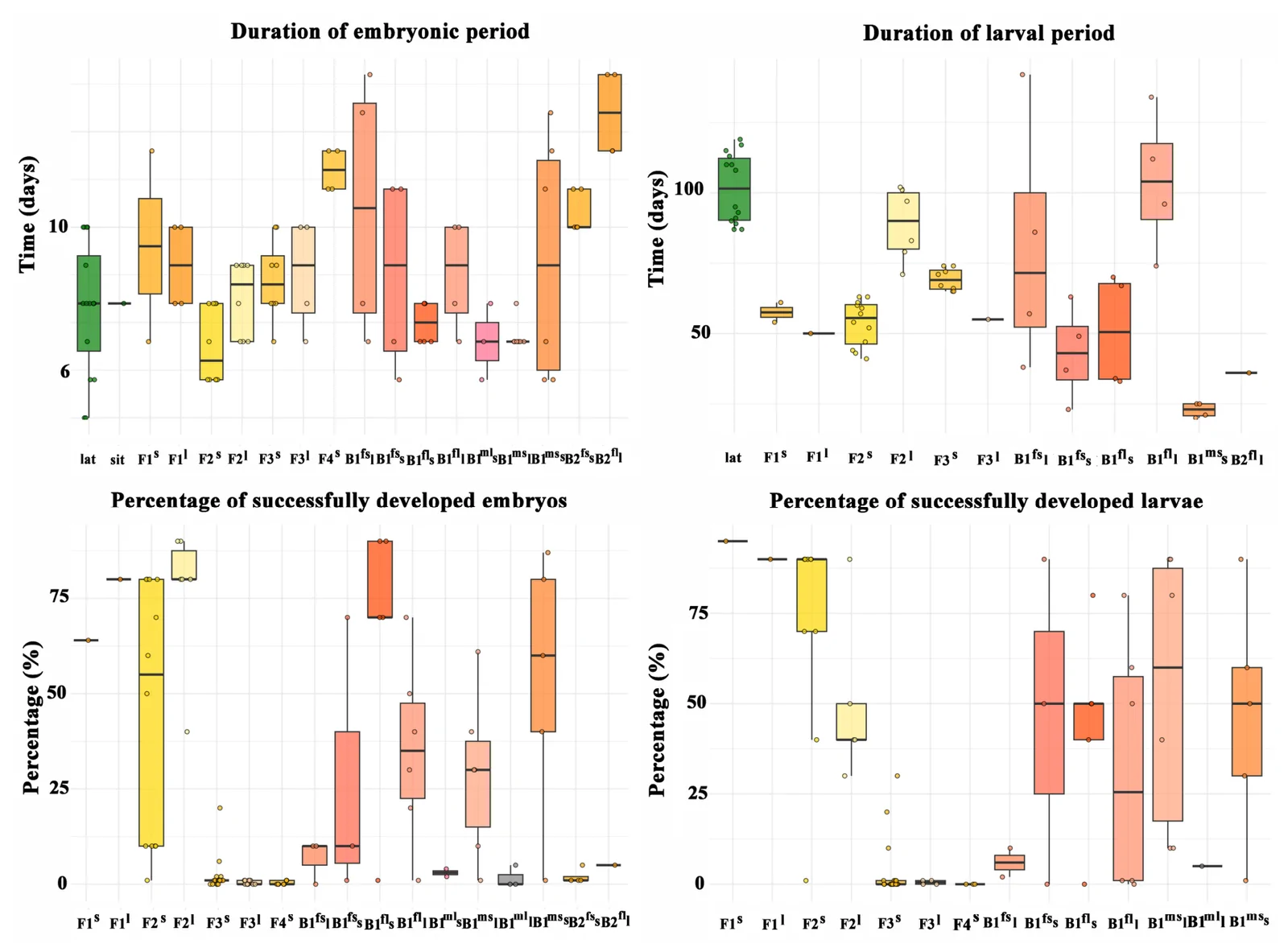
Variability of duration of embryonic and larval periods and percentage of successfully developed embryos and larvae in *Bufotes latastii, B. sitibundus*, and their hybrid offspring. Abbreviations for various types of hybrids are given as in Table 1. The color scheme is the same as in Figure 1.

**Figure 5:**
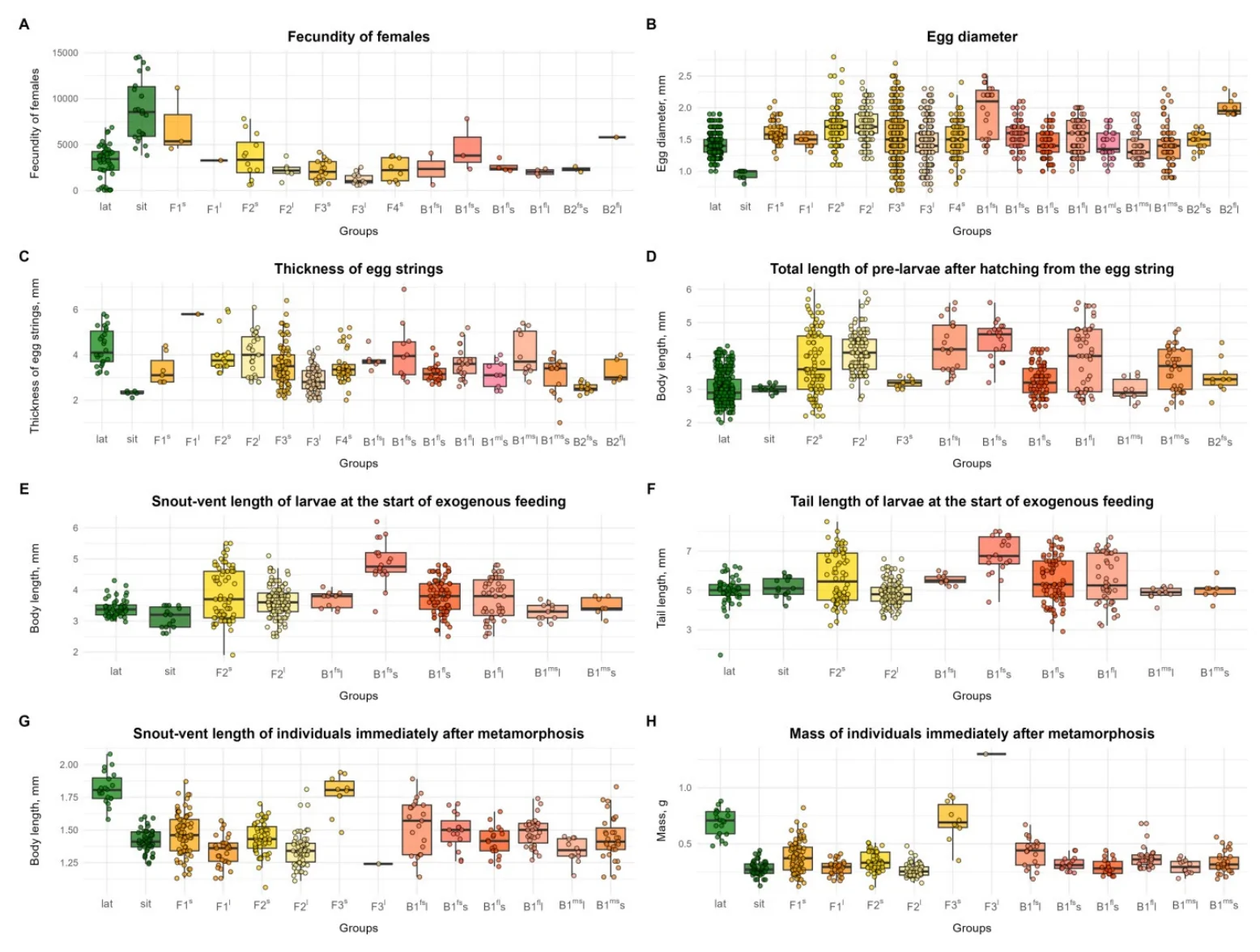
Variability of some reproductive and developmental traits in *Bufotes latastii, B. sitibundus*, and their hybrid offspring. Abbreviations for various types of hybrids are given as in Table 1. The color scheme is the same as in Figure 1.

Almost all hybrids (97%) were diploid, and only one individual was tetraploid (Table 1). The pre-larvae hatched from egg strings on days 2–5 and started exogenous feeding on days 5–7. The average duration of embryonic development from the laying by the female of the egg string until the start of active feeding of tadpoles was 9.8 days (Table 3; Figure 4), but in different individuals it can take place over a period of 7 to 12 days. The percentage of successfully hatched pre-larvae ranged from 10 to 64% and completed metamorphosis larvae was 50–95% (Table 4; Figure 4). The average duration of larval (without embryonic) development was 57.3 days (Table 3; Figure 4), but in different individuals it can take place over a period of 53 to 61 days. The average snout-vent length and mass of individuals after metamorphosis was 14.7 mm and 0.37 g, respectively (Table 3; Figure 5). The two-year-old individuals were able to reproduce. The sex ratio among these hybrids was near equal. The mean fecundity of females was 3364 eggs, the thickness of egg strings was 4.0 mm, and the diameter of eggs (vitellus) was 1.7 mm (Table 5; Figure 5).

##### Hybrids of the Second Generation (F2^s^)

In the crossing, we obtained diploid (28%), triploid (67%), and tetraploid (5%) individuals (Table 1). The pre-larvae hatched from egg strings on days 2–3 and started exogenous feeding on days 5–7. The average duration of embryonic development was 6.9 days (in different individuals, 6–8 days; Table 3; Figure 4). The percentage of successfully developed eggs obtained from twelve pairs ranged from very low (1–10%) in 40% of pairs to relatively high (50–80%) in other pairs (Table 4; Figure 4). The mean total length of pre-larvae was 3.8 mm. The average snout-vent and tail lengths of larvae at the start of exogenous feeding were 3.9 and 5.6 mm, respectively. The average duration of larval development was 53.7 days (in different individuals, 41–63 days; Table 3; Figure 4). The percentage of successfully completed metamorphosis larvae ranged from 1% to 90% (Table 4; Figure 4). The average snout-vent length and mass of individuals after metamorphosis were 14.3 mm and 0.34 g, respectively (Table 3; Figure 5). The two-year-old offspring individuals were capable of reproduction. The sex ratio was near equal. The mean fecundity of females was 2197 eggs, the thickness of egg strings was 3.8 mm, and the diameter of eggs was 1.6 mm (Table 5; Figure 5).

##### Hybrids of the Third Generation (F3^s^)

We used in our breeding experiments seventeen pairs of F2^s^ hybrids with unknown ploidy (predominantly 3*n*). Most (60%) of the resulting F3 hybrids were triploids, and other individuals (40%) were diploid (Table 1). The pre-larvae hatched from egg strings on the 4th–5th day and started exogenous feeding on the 6th–7th day. The average duration of embryonic development was 8.6 days (in different individuals, 7–10 days; Table 3; Figure 4). A relatively high percentage (20%) of developed eggs was observed in only one clutch. In the other 20 clutches, we found a very low percentage (<6%) of developed eggs (Table 4; Figure 4). The mean total length of pre-larvae was 3.2 mm. The average duration of larval development was 69.3 days (in different individuals, 65–74 days; Table 3; Figure 4). The percentage of successfully completed metamorphosis larvae in the vast majority of cases (90%) was very low (0–1%), and only in two cases it ranged from 10% to 30% (Table 4; Figure 4). The average snout-vent length and mass of individuals after metamorphosis were 17.8 mm and 0.71 g, respectively (Table 3; Figure 5). The two-year-old offspring individuals were able to reproduce. The sex ratio was near equal. The mean fecundity of females was 2270 eggs, the thickness of egg strings was 3.5 mm, and the diameter of eggs was 1.5 mm (Table 5; Figure 5).

##### Hybrids of the Fourth Generation (F4^s^)

We crossed eight pairs of F3 hybrids with unknown ploidy (predominantly 3*n*) and observed a complete absence or an extremely low (˂1%) percentage of successfully developed eggs (Table 4; Figure 4). The pre-larvae hatched from egg strings on the 5th day and started exogenous feeding on the 6th–7th day. The average duration of embryonic development was 11.5 days (in different individuals, 11–12 days; Table 3; Figure 4). Of the two clutches, only two larvae survived to metamorphosis, but neither of them was able to successfully complete it.

##### The First-Generation Backcrosses with Males of *B. latastii* (B1^fs^l)

We obtained diploid (12%), triploid (81%), and pentaploid offspring (Table 1). The average duration of embryonic development was 10.5 days (in different individuals, 7–14 days; Table 3; Figure 4). The percentage of developed eggs in three clutches did not exceed 10%, and the average survival of larvae to metamorphosis was 6% (Table 4; Figure 4). The average total length of pre-larvae was 4.3 mm. The average snout-vent and tail lengths of larvae at the start of exogenous feeding were 3.7 and 5.6 mm, respectively. The average duration of larval development was 80.8 days (in different individuals, 38–142 days; Table 3; Figure 4). The average snout-vent length and mass of individuals after metamorphosis were 15.3 mm and 0.42 g, respectively (Table 3; Figure 5). We did not conduct further experiments on raising these offspring.

##### The First-Generation Backcrosses with Males of *B. sitibundus* (B1^fs^s)

In this crossing, 17% of diploid and 83% of triploid offspring were obtained (Table 1). The average duration of embryonic development was 8.8 days (in different individuals, 6–11 days; Table 3; Figure 4). The percentage of developed eggs in three clutches was 1–70%, and the average survival of larvae to metamorphosis was 47% (Table 4; Figure 4). The mean total length of pre-larvae was 4.5 mm. The average snout-vent and tail lengths of larvae at the start of exogenous feeding were 4.9 and 6.8 mm, respectively. The average duration of larval development was 43.0 days (in different individuals, 23–63 days; Table 3; Figure 4). The average snout-vent length and mass of individuals after metamorphosis were 14.9 mm and 0.32 g, respectively (Table 3; Figure 5). The two-year-old offspring individuals were able to reproduce. The sex ratio was near equal. The mean thickness of egg strings was 2.5 mm, and the diameter of eggs was 1.5 mm (Table 5; Figure 5).

##### The First-Generation Backcrosses with Females of *B. latastii* (B1^ms^l)

All offspring were diploid (Table 1). The duration of embryonic development was seven days (Table 3; Figure 4). The percentage of successfully developed eggs ranged from 1 to 61%, and the average survival of larvae to metamorphosis was 53% (Table 4; Figure 4). The mean total length of pre-larvae was 3.0 mm. The average snout-vent and tail lengths of larvae at the start of exogenous feeding were 3.3 and 4.9 mm, respectively. The average snout-vent length and mass of individuals after metamorphosis were 13.5 mm and 0.29 g, respectively (Table 3; Figure 5). We did not conduct further experiments on raising these offspring.

##### The First-Generation Backcrosses with Females of *B. sitibundus* (B1^ms^s)

In this crossing, we obtained diploid (95%) and triploid (5%) offspring (Table 1). The average duration of embryonic development was 9.2 days (in different individuals, 6–13 days; Table 3; Figure 4). The percentage of successfully developed eggs in three clutches varied from 1 to 87%, and survival of larvae to metamorphosis was 1–90% (Table 4; Figure 4). The mean total length of pre-larvae was 3.6 mm. The average snout-vent and tail lengths of larvae at the start of exogenous feeding were 3.5 and 5.0 mm, respectively. The average duration of larval development was 22.8 days (in different individuals, 20–25 days; Table 3; Figure 4). The average snout-vent length and mass of individuals after metamorphosis were 14.4 mm and 0.33 g, respectively (Table 3; Figure 5). We did not conduct further experiments on raising these offspring.

##### The Second-Generation Backcrosses with Males of *B. sitibundus* (B2^fs^s)

A single studied individual was tetraploid (Table 1). The percentage of successfully developed embryos was extremely low (˂5%; Table 4; Figure 4). The pre-larvae hatched from egg strings on the 5th day and started exogenous feeding on the 5th–6th day. The average duration of embryonic development was 10.5 days (in different individuals, 10–11 days; Table 3; Figure 4). The average total length of pre-larvae was 3.4 mm. We did not conduct further experiments on raising these offspring.

#### 3.3.2. Hybrids Between *B. latastii* Females and *B. sitibundus* Males

##### Hybrids of the First Generation (F1^l^)

We made repeated attempts to obtain this combination of crosses, but in most cases they were unsuccessful. This result was not obtained due to the low percentage of development or high mortality of larvae, but because of strong disproportion in the size of males and females of parental species. Larger *B. sitibundus* males capture smaller *B. latastii* females in the amplexus and in most cases release them immediately. During the formation of stable amplexus, males strangled females. Only once did we successfully cross a female *B. latastii* and a male *B. sitibundus*. Almost all (98%) obtained F1 hybrids were diploid and only one individual was triploid (Table 1). The pre-larvae hatched from egg strings on days 2–4 and started exogenous feeding on day 6. The percentage of successfully hatched pre-larvae was 80%, and the percentage of larvae that completed metamorphosis was 90% (Table 4; Figure 4). The average duration of embryonic development was 9.0 days (in different individuals, 8–10 days; Table 3; Figure 4). The duration of larval development was 50 days. The average snout-vent length and mass of individuals after metamorphosis were 13.4 mm and 0.29 g, respectively (Table 3; Figure 5). The two-year-old offspring individuals were able to reproduce. The sex ratio was near equal. The mean fecundity of females was 2270 eggs, the thickness of egg strings was 3.7 mm, and the diameter of eggs was 1.6 mm (Table 5; Figure 5).

##### Hybrids of the Second Generation (F2^l^)

In this type of crossing, we obtained only triploid individuals (Table 1). The pre-larvae hatched from egg strings on days 2–3 and started exogenous feeding on days 3–5. The average duration of embryonic development was 8.1 days (in different individuals, 7–9 days; Table 3; Figure 4). The percentage of successfully developed eggs ranged from middle (40%) to very high (>90%). The mean total length of pre-larvae was 4.1 mm. The average snout-vent and tail lengths of larvae at the start of exogenous feeding were 3.6 and 4.8 mm, respectively. The average duration of larval development was 88.8 days (in different individuals, 71–102 days; Table 3; Figure 4). The percentage of successfully completed metamorphosis larvae ranged from 30% to 90% (Table 4; Figure 4). The average snout-vent length and mass of individuals after metamorphosis were 13.5 mm and 0.26 g, respectively (Table 3; Figure 5). The two-year-old offspring individuals were able to reproduce. The sex ratio was near equal. The mean fecundity of females was 1248 eggs, the thickness of egg strings was 2.9 mm, and the diameter of eggs was 1.5 mm (Table 5; Figure 5).

##### Hybrids of the Third Generation (F3^l^)

The parental F2 hybrids were fertile and can successfully breed. In five obtained clutches out of eleven, the egg development stopped before the pre-larvae hatched. In the other six clutches, the percentage of successful egg development was extremely low (˂1%). The pre-larvae hatched from egg strings on days 4–5 and started exogenous feeding on days 6–7. The average duration of embryonic development was 8.8 days (in different individuals, 7–10 days; Table 3; Figure 4). All obtained larvae except one died before metamorphosis. The duration of larval development of the remaining larva was 55 days. Its snout-vent length and mass after metamorphosis were 12.4 mm and 0.13 g, respectively (Table 3; Figure 5). This individual died shortly after metamorphosis. Its ploidy has not been studied.

##### The First-Generation Backcrosses with Males of *B. sitibundus* (B1^fl^s)

We obtained diploid (6%) and triploid (94%) offspring (Table 1). The pre-larvae hatched from egg strings on days 3–4 and started exogenous feeding on days 3–4. The average duration of embryonic development was 7.5 days (in different individuals, 7–8 days; Table 3; Figure 4). The percentage of successfully developed eggs in five clutches was 1–90%, and survival of larvae to metamorphosis was 0–80% (Table 4; Figure 4). The mean total length of pre-larvae was 3.3 mm. The average snout-vent and tail lengths of larvae at the start of exogenous feeding were 3.8 and 5.5 mm, respectively. The average duration of larval development was 51.0 days (in different individuals, 33–70 days; Table 3; Figure 4). The average snout-vent length and mass of individuals after metamorphosis were 14.1 mm and 0.29 g, respectively (Table 3; Figure 5). We did not conduct further experiments on raising these offspring.

##### The First-Generation Backcrosses with Males of *B. latastii* (B1^fl^l)

As a result of this crossing, we obtained triploid offspring (Table 1). The pre-larvae hatched from egg strings on days 4–5 and started exogenous feeding on days 3–5. The average duration of embryonic development was 8.8 days (in different individuals, 7–10 days; Table 3; Figure 4). The percentage of successfully developed eggs ranged from very low (1%) to relatively high (20–70%), and the survival rate of larvae was 38% (Table 4; Figure 4). The mean total length of pre-larvae was 4.0 mm. The average snout-vent and tail lengths of larvae at the start of exogenous feeding were 3.7 and 5.5 mm, respectively. The average duration of larval development was 104.0 days (in different individuals, 74–134 days; Table 3; Figure 4). The average snout-vent length and mass of individuals after metamorphosis were 14.8 mm and 0.38 g, respectively (Table 3; Figure 5). The two-year-old offspring individuals were able to reproduce. The sex ratio was near equal. The fecundity of the backcross female was 5190 eggs, the mean thickness of egg strings was 3.3 mm, and the diameter of eggs was 2.0 mm (Table 5; Figure 5).

##### The First-Generation Backcrosses with Females of *B. sitibundus* (B1^ml^s)

The pre-larvae hatched from egg strings on days 3–4 and started exogenous feeding on days 3–4. The average duration of embryonic development was 7.0 days (in different individuals, 6–8 days; Table 3; Figure 4). The percentage of developed eggs was 3%—the larvae did not hatch (Table 4; Figure 4).

##### The First-Generation Backcrosses with Females of *B. latastii* (B1^ml^l)

The percentage of developed eggs and larvae was very low (0–5%; Table 4; Figure 4). Only one larva began to feed, but after that it quickly died.

##### The Second-Generation Backcrosses with Males of *B. latastii* (B2^fl^l)

The percentage of successful embryonic development in this crossing was very low (5%; Table 4; Figure 4). The pre-larvae hatched from egg strings on the 5th day and started exogenous feeding on the 7th–9th day. The average duration of embryonic development was 11.0 days (in different individuals, 12–14 days; Table 3; Figure 4). The larvae did not survive to metamorphosis. The only tadpole we studied turned out to be tetraploid (Table 1).

### 3.4. Embryonic and Larval Development

#### 3.4.1. Duration of Embryonic Period

At a water temperature of 18 °C, the embryonic development of the parental species usually lasts 5–10 days (Table 3; Figure 4). Nearly the same development period was observed in most types of hybrid offspring. However, in some crosses (the first and fourth generations of hybrids of *B*. *sitibundus* ♀ × *B. latastii* ♂ (predominantly 2*n* and unknown ploidy, respectively), the B1 backcrosses (*B. sitibundus* ♀ × *B. latastii* ♂) ♀ × *B. latastii* ♂ (predominantly 2*n*), and the B1 backcrosses *B. sitibundus* ♀ × (*B. sitibundus* ♀ × *B. latastii* ♂) ♂ (predominantly 2*n*)), the duration of embryonic development was significantly longer (up to 14 days; Table 3; Figure 4).

#### 3.4.2. Percentage of Successfully Developed Pre-Larvae and Larvae

The one-way ANOVA showed significant differences between various hybrid categories for both pre-larvae (F = 121.9, df = 1.0, *p* < 0.001) and larvae (F = 484.5, df = 1.0, *p* < 0.001). Based on these parameters, two groups can obviously be distinguished (Figure 4). The first group includes hybrids that on average have a relatively high percentage (>20%) of successful development for both pre-larvae and larvae. This group comprised the F1 and F2 hybrids, and the backcrosses (*B. latastii* ♀ × *B. sitibundus* ♂) females or (*B. sitibundus* ♀ × *B. latastii* ♂) males on any parental species (Table 4). In the last crosses, almost no specimen survived.

#### 3.4.3. Total Length of Pre-Larvae

The one-way ANOVA showed significant differences in total length of pre-larvae (immediately after hatching from the egg string) among parental species and various hybrid categories (F = 8123.8, df = 1.0, *p* < 0.001). It is important to note that the parental species do not differ in this parameter, while most hybrids have longer pre-larvae, and in many cases these differences are significant. The longest pre-larvae were observed in the second-generation hybrids of *B. latastii* ♀ × *B. sitibundus* ♂, the B1 backcrosses (*B. sitibundus* ♀ × *B. latastii* ♂) females on *B. latastii* or *B. sitibundus* males (Table 3). In addition, in many hybrids the variability in this parameter is noticeably higher than in the parental species (Figure 4).

#### 3.4.4. Duration of Larval Period

At a water temperature of 18 °C, the larval development of the parental species usually lasts 87–120 days (Table 3; Figure 4). However, the majority of hybrid types (except the second-generation hybrids of *B. latastii* ♀ × *B. sitibundus* ♂ and the backcross (*B. latastii* ♀ × *B. sitibundus* ♂) ♀ × *B. latastii* ♂) have a much shorter larval period.

#### 3.4.5. Snout-Vent (SVL) and Tail (TL) Length of Larvae at the Start of Exogenous Feeding

The one-way ANOVA showed significant differences for both SVL (F = 7809.6, df = 1.0, *p* < 0.001) and TL (F = 6057.5, df = 1.0, *p* < 0.001) among parental species and various hybrid categories. The parental species do not differ in these parameters (Figure 4). The average SVL and TL of the B1 backcrosses (*B. sitibundus* ♀ × *B. latastii* ♂) ♀ × *B. sitibundus* ♂ were significantly longer (4.9 vs. 3.1–3.9 mm and 6.8 vs. 4.8–5.6 mm, respectively; Table 3) than in parental species and all other categories of hybrids (*p* < 0.05; except TL for the backcrosses (*B. sitibundus* ♀ × *B. latastii* ♂) ♀ × *B. latastii* ♂). In addition, in some hybrids the variability in this parameter is noticeably higher than in parental species (Figure 4).

#### 3.4.6. Snout-Vent Length and Mass of Individuals After Metamorphosis

The one-way ANOVA showed significant differences in SVL (F = 11,997.9, df = 1.0, *p* < 0.001) and mass (F = 1439.7, df = 1.0, *p* < 0.001) of individuals immediately after metamorphosis among the parental *B. latastii* and various hybrid categories. Based on these parameters, two groups can obviously be distinguished (Figure 4; Table 3). The first group includes *B. latastii* and the third-generation hybrids of *B. sitibundus* ♀ × *B. latastii* ♂, which were characterized by significantly (*p* < 0.05) higher SVL (1.8 vs. 1.2–1.5 cm) and mass (0.7 vs. 0.3–0.4 g) in metamorphs. The second group includes all other hybrid types studied.

### 3.5. Reproductive Characteristics of Parental and Hybrid Females

#### 3.5.1. Female Fecundity

The one-way ANOVA showed significant differences in female fecundity among parental species and various hybrid categories (F = 268.0, df = 1.0, *p* < 0.001). *B. sitibundus* significantly differed (*p* < 0.001) from *B. latastii* and all categories of hybrids. Larger females of *B*. *sitibundus* tend to lay more eggs (average 8583) than smaller *B. latastii* and hybrids (1248–3364; Figure 4 and Table 3). Among hybrid females, the one-way ANOVA also showed significant differences (F = 153.3, df = 1.0, *p* < 0.001). The F2 hybrids obtained from crossings with *B. latastii* females laid significantly (*p* ˂ 0.01) fewer eggs than the F1 hybrids obtained from crossings with *B. sitibundus* females (1248 vs. 3364). We found no correlation between fecundity of hybrid females and occurrence of polyploids in each type of crossing (*r*^2^ = 0.009, *n* = 6, F = 0.04, df = 1.4, *p* = 0.860).

#### 3.5.2. Thickness of Egg Strings

The one-way ANOVA showed significant differences among parental species and various categories of hybrids (F = 3361.8, df = 1.0, *p* < 0.001). *B. latastii* significantly differed (*p* < 0.05) from *B. sitibundus* and most categories of hybrids (except the F1 hybrids of *B. sitibundus* ♀ × *B. latastii* ♂ and the B1 backcrosses (*B. latastii* ♀ × *B. sitibundus* ♂) ♀ × *B. latastii* ♂). In its turn, *B. sitibundus* significantly (*p* < 0.01) differed from the F1 hybrids of *B. sitibundus* ♀ × *B. latastii* ♂. Females of *B. latastii* lay thicker egg strings (average 4.3 mm) than *B. sitibundus* (3.1 mm; Figure 4 and Table 3). Among studied hybrids and backcrosses, the one-way ANOVA also showed significant differences (F = 1375.3, df = 1.0, *p* < 0.001). Both types of F1 hybrids, as well as the F2 hybrids *B. sitibundus* ♀ × *B. latastii* ♂, significantly differed (*p* < 0.05) from the F2 hybrid *B. latastii* ♀ × *B. sitibundus* ♂ and the B1 backcrosses (*B. sitibundus* ♀ × *B. latastii* ♂) ♀ × *B. sitibundus* ♂ (3.6–4.0 vs. 2.5–2.9 mm). The F2 hybrids *B. latastii* ♀ × *B. sitibundus* ♂ significantly differed (*p* < 0.05) from all other types of studied hybrids (2.9 vs. 3.5–4.0 mm; Figure 5). We found no significant correlation between the thickness of egg strings and the occurrence of polyploids among hybrids of each type of cross (*r*^2^ = 0.487, *n* = 7, F = 4.75, df = 1.5, *p* = 0.081).

#### 3.5.3. Egg (Vitellus) Diameter

The one-way ANOVA showed significant differences among parental species and various categories of hybrids (F = 12,261.1, df = 1.0, *p* < 0.001). The post hoc comparisons revealed that *B. latastii* and *B. sitibundus* did not differ from each other but significantly differ (*p* < 0.05) from some hybrid types (both F1 hybrids, the F2 hybrid *B. sitibundus* ♀ × *B. latastii* ♂, and the B1 backcross (*B. latastii* ♀ × *B. sitibundus* ♂) ♀ × *B. latastii* ♂). Females of parental species produced smaller eggs (average 1.4 mm) than such hybrids (1.6–2.0 mm; Figure 5 and Table 3). Among hybrids and backcrosses, the one-way ANOVA also showed significant differences (F = 6578.4, df = 1.0, *p* < 0.001). The F1 hybrids *B. sitibundus* ♀ × *B. latastii* ♂ significantly differed (*p* < 0.01) from the F2 and F3 hybrids (1.7 vs. 1.5–1.6 mm). The F1 hybrids *B. latastii* ♀ × *B. sitibundus* ♂ significantly differed (*p* < 0.05) from the F2 hybrids of *B. latastii* ♀ × *B. sitibundus* ♂ and the B1 backcrosses (*B. latastii* ♀ × *B. sitibundus* ♂) ♀ × *B. latastii* ♂ (1.6 vs. 1.5 and 2.0 mm, respectively). The B1 backcrosses (*B. latastii* ♀ × *B. sitibundus* ♂) ♀ × *B. latastii* ♂ significantly differed (*p* < 0.05) from other types of hybrids and backcrosses with the exception of the F1 hybrid of *B. sitibundus* ♀ × *B. latastii* ♂ (2.0 vs. 1.5–1.6 mm; Figure 4). We found no significant correlation between egg diameter and occurrence of polyploids in hybrids of each type of crossing (*r^2^* = 0.000, *n* = 7, F = 0.00, df = 1.5, *p* = 0.825).

## 4. Discussion

Our study has established that hybridization between phylogenetically distant species (different subgenera) of the genus *Bufotes* results in the appearance of polyploid hybrids. Even among F1 hybrids, single polyploid individuals may appear. The presence of triploid individuals among natural hybrids of diploid amphibian species has also been reported previously [64,65,66]. In some cases, anurans produce unreduced gametes [67], which could have led to the emergence of polyploid species that arose independently in several families of anurans [32]. Hybrids may use clonal or hemiclonal methods of reproduction, in which one or all of the parental genomes are passed on to the offspring without recombination [14,68,69]. However, some hybrids retain a strong connection with parent species, which is necessary for successful reproduction [14].

Laboratory crosses between various amphibian species have been conducted repeatedly (e.g., [67,70]). Previous crosses of toads of the family Bufonidae Gray, 1825 [67,71,72,73,74,75,76,77,78], have revealed the following pattern: when crossing more closely related species, fewer polyploid F1 offspring are born, and these offspring reach later stages of development compared to crossing genetically more divergent species. When crossing *Bufotes* with representatives of various genera (*Anaxyrus* Tschudi, 1845, *Incilius* Cope, 1863, *Rhinella* Fitzinger, 1826, *Epidalea* Cope, 1864, *Bufo* Garsault, 1764, and *Duttaphrynus* Frost et al., 2006), in F1 offspring, in addition to diploid hybrids, triploid and pentaploid individuals were also observed [14,73,78]. Moreover, when representatives of the genus *Bufotes* were hybridized with *Incilius valliceps* (Wiegmann, 1833) and some *Anaxyrus* species, all hybrid offspring were triploid, whereas when hybridized with *Rhinella horribilis* (Wiegmann, 1833) and some *Sclerophrys* Tschudi, 1838, species, they were pentaploid [14]. Based on obtained data, Bogart [73] made several conclusions. A small percentage of eggs of parental species can be diploid or tetraploid, which is probably the result of suppressed maturation divisions in some oocytes. Triploid and pentaploid hybrids result from fertilization by haploid sperm of such oocytes. If polyspermy and tetraploid tadpoles do occur, it must be very rare.

In our experiments, triploids (2% in the cross *B*. *latastii* ♀ × *B*. *sitibundus* ♂; Table 1) were sometimes found among F1 hybrids, which may indicate the possibility of the formation of diploid oocytes in females of parental species by the fusion of the haploid second polar body with the haploid female pronucleus. Theoretically, the formation of diploid spermatozoa is also possible, since all tetraploid species of this genus are characterized by such gametes. In natural populations of various amphibians, several cases of the appearance of diploid gametes can also be observed, which can lead to the appearance of autotriploids [32].

The presence of tetraploid tadpoles among the F1 hybrids *B*. *sitibundus* ♀ × *B*. *latastii* ♂ (3%; Table 1) can be explained by the fusion of the diploid first polar body with the haploid female pronucleus (oocytes with ploidy 3*n*). Previously, Kawamura and Moriwaki [79] obtained a tetraploid tadpole of *Fejervarya kawamurai* Tjong, Matsui, Kuramoto, Nishioka, et Sumida, 2011. Theoretically, the appearance of polyploid embryos may also be a consequence of pathological polyspermy. However, it should be noted that, as a rule, polyspermy does not lead to an increase in ploidy of offspring [80].

In our experiments, pentaploid progeny were observed in B1 backcross ((*B*. *sitibundus* ♀ × *B*. *latastii* ♂) ♀ × *B*. *latastii* ♂), as well as hexaploid progeny were found among F3 hybrids *B*. *sitibundus* ♀ × *B*. *latastii* ♂ (Table 1). Previously, pentaploid and hexaploid tadpoles were obtained in crosses between species of the genus *Bufo* [74]. This may indicate that in polyploid females the release of the second-division polar bodies is suppressed and, therefore, fusion of the second diploid polar body with the diploid female pronucleus occurs. This promotes the formation of tetraploid oocytes, which, when fertilized with haploid or diploid sperm, can produce pentaploid and hexaploid offspring, respectively.

Some differences in results of previously published data [73,74] and ours are most likely explained by significant differences in the divergence time between representatives of different subgenera within the genus *Bufotes* (about 16.5 million years [46]) and between *Bufotes* and various genera of the family Bufonidae (24.6–29.0 million years [81]). Among F1 hybrids obtained by crossing representatives of various genera with species of the genus *Bufotes*, polyploidy in the offspring was observed much more often than between species within the genus *Bufotes*. Pentaploids were completely absent among the F1 hybrids we obtained (only found among backcrosses). This trend is likely a consequence of increasing reproductive (postzygotic) isolation in amphibians with increasing genetic distance between parental species [78,82,83].

**Figure 6:**
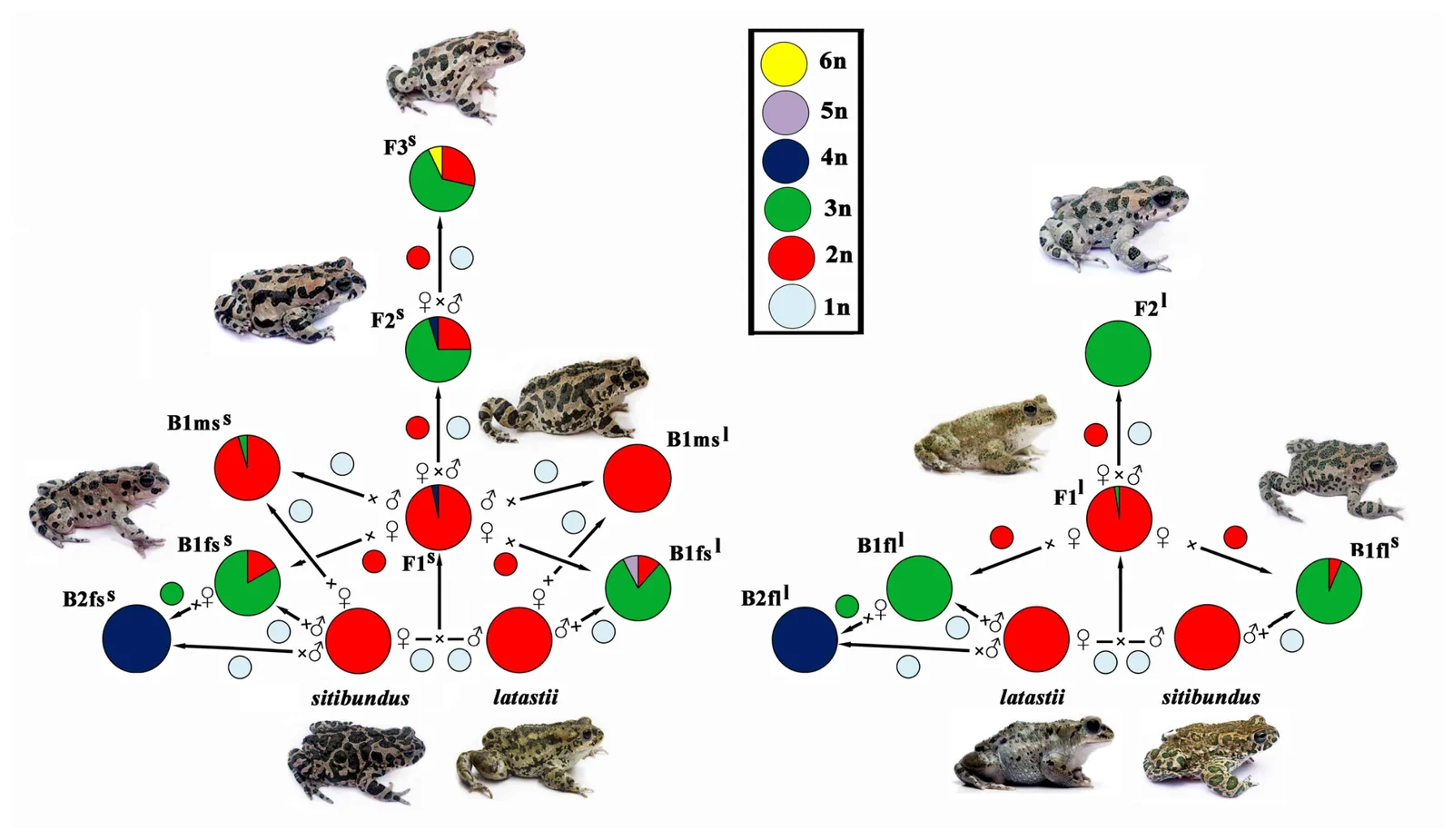
Schematic diagram of hybridization experiments between *Bufotes latastii* and *B. sitibundus*. Different colors indicate the ploidy level of the offspring (large circles) and the expected ploidy of gametes (small circles). Abbreviations for various types of hybrids are given as in Table 1.

In our crossing experiments, the number of polyploid individuals in the second and subsequent generations of hybrids was significantly higher compared to F1 hybrids. For example, in F2 hybrids between females of *B*. *latastii* and males of *B*. *sitibundus*, all offspring were triploid (Table 1; Figure 6). These data indicate that in nature, crosses between diploid species can lead to the emergence of polyploid individuals, which is consistent with the basic principles of the reticulate evolution [32,34]. However, it is important to note that when crossing first-, second-, and subsequent-generation hybrids, the survival rate of the offspring decreases significantly (Table 4; Figure 4). Therefore, it is undoubtedly not the second- and subsequent-generation hybrids that play the greatest role in reticular speciation but rather backcrosses, the survival rate of which is usually somewhat higher. Moreover, crossing of hybrids with parental species results in the appearance of polyploid offspring, which, upon further crossing with parental species, results in the appearance of mixed diploid–polyploid and even fully polyploid hybrid lineages. For example, in our experiments, when crossing females of F1 hybrid *B*. *sitibundus* ♀ × *B*. *latastii* ♂ with males of any parental species, the majority (81–100%; Table 1) of the offspring were triploid. In subsequent hybrid crosses, we also observed polyploid offspring (Figure 6).

In the case of the genus *Bufotes*, crosses of hybrid females between *B*. *sitibundus* ♀ (and closely related species) and *B. latastii* ♂ with *B. latastii* males appear to have resulted in natural triploid species. Initially, in the early stages of the formation of these triploid species, both parental species and their hybrids were syntopic. Then, with changing environmental conditions in most of Central Asia (perhaps, aridization in the Pleistocene [53]), a moisture-loving *B. latastii* became extinct on vast territories. The triploid lineage could continue to coexist with the more drought-tolerant parent species (*B. perrini* Mazepa, Litvinchuk, Jablonski et Dufresnes, 2019, *B. sitibundus*, and *B. turanensis* (Hemmer, Schmidtler et Boehme, 1978)) without interacting with them during the reproduction process. Similar populations (undescribed triploid species and diploid *B. perrini*) were found in northeastern Iran [46,84,85]. Hybrid heterosis and increased resistance of polyploids to environmental changes [6] allowed them to occupy previously vacant ecological niches (as a rule, mountains), where modern triploid species subsequently formed.

How do obtained laboratory triploid hybrids reproduce? In most known diploid–polyploid hybrid complexes among vertebrates, triploid hybrids reproduce by clonal or hemiclonal modes of reproduction, such as gynogenesis, kleptogenesis, parthenogenesis, and others [14,86]. However, it is important to note that among anurans in hybrid complexes, such as those of water frogs of the genus *Pelophylax* Fitzinger, 1843, triploid hybrid females (*P. esculentus* Linnaeus, 1758) typically produce haploid gametes rather than diploid ones, resulting in diploid offspring [87,88]. In the process of evolution, various hybrid complexes of vertebrates independently developed different mechanisms, leading to the emergence and maintenance of triploid hybrids [14]. Green toads of the genus *Bufotes* exhibit their own unusual reproductive method, in which triploids can reproduce successfully by crossing females producing diploid oocytes with males producing haploid sperm. This makes the existence of natural bisexual triploid species possible [37,69].

Our experiments did not provide a definitive answer to the question of whether laboratory triploid hybrids are capable of producing only triploid offspring. The only thing we can say with certainty is that diploid hybrids, when crossed, usually produce triploid offspring (Figure 6). For example, when F1 hybrids (*B. latastii* females and *B. sitibundus* males) were crossed, all their F2 offspring were triploid (Table 1). This means that males typically produce haploid sperm, while females produce diploid oocytes. When predominantly (67%) triploid F2 hybrids between *B. sitibundus* females and *B. latastii* males were crossed with each other, most of the offspring were also triploid (60%; Figure 6). This allows us to state with a reasonable degree of confidence that laboratory triploids can produce triploids by hemiclonal reproduction, similar to natural triploid species.

As for the origin of tetraploid species, it cannot be ruled out that such species originated from triploid ones. For example, in lizards of genera *Aspidoscelis* Fitzinger and *Darevskia* Arribas 1999, triploid parthenogenetic females can cross with males of diploid bisexual syntopic species, which can result in tetraploid individuals [89,90]. However, based on our available data, it can be assumed that tetraploids can arise directly as a result of crossing parental diploid species, bypassing the stage of triploid species. For example, tetraploids were occasionally found in offspring of female *B. sitibundus* and male *B. latastii* (Table 1). They were also found among the progeny of F1 hybrids obtained by crossing *B. sitibundus* females and *B. latastii* males, as well as among second-generation backcrosses (Table 1; Figure 6). However, based on results of our experiments we cannot answer the question of whether our laboratory tetraploid hybrids can produce tetraploid offspring.

It is unclear what type of sex inheritance the parent species use. Species of the genus *Bufotes* exhibit both of the most common types in anurans. In various populations of *B. viridis*, both male- and female-heterogametic systems (XX ♀/XY ♂ and ZW ♀/ZZ ♂) were observed [91,92]. In our experiments, F1 hybrids were bisexual with approximately the same sex ratio, indicating a similar inheritance system in both parental species. However, we do not know exactly what type of sex inheritance they had. This question will be addressed in future research. F2–F3 hybrids and backcrosses were represented by both sexes as well.

Some authors [74] indicate that the size of eggs in toads of the family Bufonidae correlate with the ploidy of oocytes. Furthermore, it has been shown that tetraploid species of green toads of the genus *Bufotes* are characterized by significantly larger eggs (vitellus + jelly) compared to diploid species (on average 4.0 mm in tetraploid *B. oblongus* vs. 2.3 mm in diploid *B*. *viridis* [93]). Our comparisons showed that the size of vitellus from diploid parental species was significantly smaller than eggs from some crosses (Table 5; Figure 5). We cannot accurately predict the ploidy of the female pronucleus based on egg diameter, as haploid egg size varies greatly even among parental species. However, assuming that average egg volume increases correspondingly with ploidy, we can calculate the change in egg diameter at different ploidy levels. In this case, the average diameter of a haploid egg would be 1.4 mm (average for parental species), a diploid egg 1.8 mm, and a triploid egg 2.0 mm.

Our data show that some hybrids are characterized by a predominance of large eggs with 2.0 mm or more in diameter (likely due to the polyploid female pronucleus). Thus, females from two crosses (B1 (*sitibundus* ♀ × *latastii* ♂) ♀ × *latastii* ♂ and B2 ((*latastii* ♀ × *sitibundus* ♂) ♀ × *latastii* ♂)) laid usually (50% or more) large eggs (Table 5). Some other hybrid females, although they did lay a small number of large eggs (8–17%), had, on average, larger eggs than parental species (average 1.5–1.7 vs. 1.0–1.4 mm; Table 5; Figure 5), indicating a presence of diploid oocytes among them. This is also confirmed by the fact that females of the F1 and F2 hybrids, which lay eggs with an average diameter of 1.5–1.7 mm, produced predominantly triploid offspring (64–100%; Table 1). However, B1 backcrosses laid eggs with the same average diameter (1.6 mm), but their offspring were tetraploid (Figure 6), likely indicating that even such relatively small oocytes can have a triploid female pronucleus.

What was the survival rate of the offspring of hybrids? Previous laboratory crossing data indicate that polyploid offspring have higher survival rates than diploid hybrids [14]. Based on the results of our study, we did not reveal such a pattern. A significant relationship between the occurrence of polyploids and the survival of offspring at embryonic and larval stages of development has not been found (*r* = −0.268, df = 1.11, *p* = 0.376 and *r* = −0.473, df = 1.90, *p* = 0.142, respectively). It should be noted, however, that embryo and larval survival rates were comparatively high in the predominantly triploid offspring obtained from crossing F1 hybrids and some of them with parental species (Table 4; Figure 4). In most other cases, survival of offspring was very low.

## 5. Conclusions

Results of our research demonstrate that hybridization between phylogenetically distant species of the genus *Bufotes* (belonging to different subgenera) leads to the formation of polyploid hybrids, including triploid, tetraploid, pentaploid, and hexaploid individuals. The appearance of polyploid individuals among first-generation (F1) hybrids, as well as in subsequent generations and backcrosses, indicates that the mechanisms underlying polyploidization operate at the earliest stages of hybrid formation. These data support the hypothesis that natural polyploid green toad species may have originated through the hybridization of diploid parental species.

Our experiments showed that crossing diploid hybrids yields predominantly triploid offspring. These triploid hybrids are capable of producing triploid offspring via hemiclonal reproduction. This mode of reproduction—in which females produce diploid oocytes and males produce haploid spermatozoa—appears to be a primary mechanism for maintaining triploid lineages of green toads in nature. In our experiments, the survival rate of polyploid offspring was not significantly higher than that of diploid hybrids. This suggests that the establishment of polyploid lineages in nature may depend not on a survival advantage for triploids, but rather on differences in ecological preferences and the degree of reproductive isolation from parental species.

The appearance of tetraploid individuals in our experiments—occurring both in direct crosses between diploid species and in crosses between hybrids—indicates that tetraploid species can arise directly from diploid ancestors, bypassing the triploid stage. However, further research is needed to determine whether laboratory-reared tetraploid hybrids are capable of producing tetraploid offspring and establishing stable lineages.

Overall, our study provides experimental evidence supporting the reticulate evolution model in the genus *Bufotes*, wherein hybridization between divergent diploid species gives rise to polyploid individuals capable of originating new polyploid species. The capacity of triploid hybrids for hemiclonal reproduction, combined with heterosis and enhanced resilience to abrupt environmental changes, likely contributed to the diversification of polyploid green toad species in Central Asia and adjacent regions. Future research should focus on the genetic mechanisms of sex determination, the genomic composition of polyploid hybrids, and the ecological factors facilitating the coexistence and divergence of polyploid lineages in natural populations.

## Figures and Tables

**Table 1:** Percentage of diploid (2*n*), triploid (3*n*), tetraploid (4*n*), and pentaploid (5*n*) offspring obtained in laboratory crossings between diploid green toads *Bufotes latastii* and *B. sitibundus*. *n* is the number of individuals for which we studied the nuclear DNA content. ♀ is female and ♂ is male.

Type of Crossing		*n*	Percentage			
			2*n*	3*n*	4*n*	5*n*
F1^s^	*sitibundus* ♀ × *latastii* ♂	29	96.6	0	3.4	0
F1^l^	*latastii* ♀ × *sitibundus* ♂	42	97.6	2.4	0	0
F2^s^	F2 (*sitibundus* ♀ × *latastii* ♂)	58	27.6	67.2	5.2	0
F2^l^	F2 (*latastii* ♀ × *sitibundus* ♂)	16	0	100	0	0
F3^s^	F3 (*sitibundus* ♀ × *latastii* ♂)	10	40.0	60.0	0	0
B1^fs^l	(*sitibundus* ♀ × *latastii* ♂) ♀ × *latastii* ♂	26	11.5	80.8	0	7.7
B1^fs^s	(*sitibundus* ♀ × *latastii* ♂) ♀ × *sitibundus* ♂	18	16.7	83.3	0	0
B1^fl^s	(*latastii* ♀ × *sitibundus* ♂) ♀ × *sitibundus* ♂	32	6.3	93.7	0	0
B1^fl^l	(*latastii* ♀ × *sitibundus* ♂) ♀ × *latastii* ♂	8	0	100	0	0
B1^ms^l	*latastii* ♀ × (*sitibundus* ♀ × *latastii* ♂) ♂	19	100	0	0	0
B1^ms^s	*sitibundus* ♀ × (*sitibundus* ♀ × *latastii* ♂) ♂	22	95.5	4.5	0	0
B2^fs^s	B2 ((*sitibundus* ♀ × *latastii* ♂) ♀ × *sitibundus* ♂)	1	0	0	100	0
B2^fl^l	B2 ((*latastii* ♀ × *sitibundus* ♂) ♀ × *latastii* ♂)	1	0	0	100	0

**Table 2:** The nuclear DNA content (pg), ploidy, and presumed combinations of genomes in hybrid offspring obtained in various laboratory crossings between diploid green toads *Bufotes latastii* and *B. sitibundus*. *n* is the number of individuals, L is the *B. latastii* genome, S is the *B. sitibundus* genome, R is the recombinant genome, and [S] is the genome of *B. sitibundus*, which might clonally transmit to offspring.

Type of Crossing	Ploidy	Genomes	The Nuclear DNA Content		
			*n*	Mean ± SD	Range
*latastii*	2*n*	LL	24	11.48 ± 0.09	11.26–11.63
*sitibundus*	2*n*	SS	4	9.48 ± 0.05	9.43–9.55
F1^s^	2*n*	LS	28	10.56 ± 0.14	10.20–10.74
F1^s^	4*n*	LLSS	1	20.78	–
F1^l^	2*n*	LS	41	10.50 ± 0.11	10.22–10.72
F1^l^	3*n*	LL[S]	1	15.47	–
F2^s^	2*n*	RR	16	10.68 ± 0.42	9.89–11.37
F2^s^	3*n*	LSR	39	15.83 ± 0.22	15.40–16.26
F2^s^	4*n*	LLSS	3	21.04 ± 0.15	20.89–21.20
F2^l^	3*n*	LSR	16	15.65 ± 0.17	15.42–16.06
F3^s^	2*n*	RR	4	9.84 ± 0.46	9.50–10.52
F3^s^	3*n*	LSR	6	15.99 ± 0.77	15.37–17.31
B1^fs^l	2*n*	LR	3	11.06 ± 0.42	10.79–11.55
B1^fs^l	3*n*	LL[S]	21	16.15 ± 0.20	15.61–16.43
B1^fs^l	5*n*	LLLSS	2	26.58 ± 0.78	26.03–27.14
B1^fs^s	2*n*	RS	3	10.13 ± 0.26	9.84–10.30
B1^fs^s	3*n*	LSS	15	15.30 ± 0.15	14.87–15.48
B1^fl^s	2*n*	RS	2	9.76 ± 0.63	9.31–10.20
B1^fl^s	3*n*	LSS	30	15.30 ± 0.37	14.68–16.47
B1^fl^l	3*n*	LL[S]	8	16.35 ± 0.29	15.68–16.57
B1^ms^l	2*n*	RS	19	10.97 ± 0.14	10.70–11.25
B1^ms^s	2*n*	RS	21	9.93 ± 0.31	9.44–10.87
B1^ms^s	3*n*	LSS	1	15.07	–
B2^fs^s	4*n*	LLLS	1	22.59	–
B2^fl^l	4*n*	LLSS	1	19.88	–

**Table 3:** Parameters (mean ± SD, range) of embryonic and larval development of *Bufotes latastii*, *B. sitibundus*, and their hybrids. *n* is the number of individuals; PLTL is total length of pre-larvae (mm); SVLL is snout-vent length of larvae at the start of exogenous feeding (mm); TL is tail length of larvae at the start of exogenous feeding (mm); DE and DL are duration of embryogenesis before the start of exogenous feeding and larval development, respectively (days); SVLM and MM are snout-vent length (cm) and mass (g) of individuals after metamorphosis, respectively.

Type of Crossing	PLTL	SVLL	TL	DE	DL	SVLM	MM
*latastii*	3.0 ± 0.5 2.0–4.3 (*n* = 312)	3.4 ± 0.3 3.0–4.3 (*n* = 52)	5.0 ± 0.6 3.7–6.3 (*n* = 51)	7.9 ± 1.7 5.0–10.0 (*n* = 7)	102.4 ± 12.6 87.0–119.0 (*n* = 7)	1.81 ± 0.13 1.58–2.08 (*n* = 21)	0.68 ± 0.14 0.36–0.88 (*n* = 21)
*sitibundus*	3.0 ± 0.1 2.8–3.2 (*n* = 10)	3.1 ± 0.3 2.6–3.5 (*n* = 15)	5.1 ± 0.5 4.2–5.9 (*n* = 15)	8.0 (*n* = 1)	–	1.42 ± 0.09 1.24–1.60 (*n* = 46)	0.28 ± 0.07 0.12–0.44 (*n* = 46)
F1^s^	–	–	–	9.8 ± 2.6 7.0–12.0 (*n* = 2)	57.3 ± 4.3 53.0–61.0 (*n* = 2)	1.47 ± 0.16 1.13–1.87 (*n* = 78)	0.37 ± 0.13 0.12–0.70 (*n* = 77)
F1^l^	–	–	–	9.0 ± 1.1 8.0–10.0 (*n* = 1)	50.0 (*n* = 1)	1.34 ± 0.11 1.13–1.57 (*n* = 30)	0.29 ± 0.07 0.17–0.41 (*n* = 30)
F2^s^	3.8 ± 1.0 2.2–6.0 (*n* = 93)	3.9 ± 0.8 1.9–5.5 (*n* = 73)	5.6 ± 1.3 3.2–8.5 (*n* = 72)	6.9 ± 0.9 6.0–8.0 (*n* = 5)	53.7 ± 8.1 41.0–63.0 (*n* = 6)	1.43 ± 0.12 1.06–1.70 (*n* = 55)	0.34 ± 0.09 0.11–0.51 (*n* = 55)
F2^l^	4.1 ± 0.6 2.7–5.9 (*n* = 115)	3.6 ± 0.5 2.5–5.1 (*n* = 115)	4.8 ± 0.6 3.6–6.6 (*n* = 115)	8.1 ± 0.9 7.0–9.0 (*n* = 4)	88.8 ± 12.9 71.0–102.0 (*n* = 3)	1.35 ± 0.13 1.11–1.81 (*n* = 58)	0.27 ± 0.06 0.15–0.48 (*n* = 58)
F3^s^	3.2 ± 0.1 3.0–3.4 (*n* = 10)	–	–	8.6 ± 1.1 7.0–10.0 (*n* = 4)	69.3 ± 3.9 65.0–74.0 (*n* = 4)	1.78 ± 0.15 1.48–1.94 (*n* = 10)	0.71 ± 0.18 0.35–0.93 (*n* = 10)
F3^l^	–	–	–	8.8 ± 1.5 7.0–10.0 (*n* = 2)	55 (*n* = 1)	1.24 (*n* = 1)	0.13 (*n* = 1)
F4^s^	–	–	–	11.5 ± 0.6 11.0–12.0 (*n* = 2)	no one survived	–	–
B1^fs^l	4.3 ± 0.8 3.2–5.6 (*n* = 20)	3.7 ± 0.3 3.3–4.1 (*n* = 10)	5.6 ± 0.3 5.2–6.0 (*n* = 10)	10.5 ± 3.5 7.0–14.0 (*n* = 2)	80.8 ± 45.3 38.0–142.0 (*n* = 2)	1.53 ± 0.21 1.14–1.89 (*n* = 21)	0.42 ± 0.13 0.19–0.67 (*n* = 21)
B1^fs^s	4.5 ± 0.6 3.2–5.6 (*n* = 20)	4.9 ± 0.7 3.3–6.2 (*n* = 20)	6.8 ± 1.0 4.4–8.0 (*n* = 20)	8.8 ± 2.6 6.0–11.0 (*n* = 2)	43.0 ± 17.09 23.0–63.0 (*n* = 2)	1.49 ± 0.14 1.26–1.70 (*n* = 15)	0.32 ± 0.06 0.24–0.44 (*n* = 15)
B1^fl^s	3.3 ± 0.5 2.4–4.2 (*n* = 80)	3.8 ± 0.6 2.5–4.8 (*n* = 80)	5.5 ± 1.1 2.9–7.7 (*n* = 80)	7.5 ± 0.5 7.0–8.0 (*n* = 3)	51.0 ± 20.2 33.0–70.0 (*n* = 2)	1.41 ± 0.11 1.22–1.64 (*n* = 18)	0.29 ± 0.07 0.21–0.44 (*n* = 18)
B1^fl^l	4.0 ± 1.0 2.6–5.6 (*n* = 50)	3.7 ± 0.7 2.5–4.8 (*n* = 48)	5.5 ± 1.2 3.2–7.7 (*n* = 48)	8.8 ± 1.5 7.0–10.0 (*n* = 2)	104.0 ± 25.3 74–134 (*n* = 1)	1.48 ± 0.11 1.27–1.74 (*n* = 31)	0.38 ± 0.10 0.27–0.68 (*n* = 31)
B1^ms^l	3.0 ± 0.3 2.5–3.5 (*n* = 10)	3.3 ± 0.3 2.9–3.7 (*n* = 11)	4.9 ± 0.3 4.1–5.2 (*n* = 11)	7.1 ± 0.4 7.0–8.0 (*n* = 1)	–	1.35 ± 0.09 1.15–1.45 (*n* = 12)	0.29 ± 0.06 0.19–0.39 (*n* = 12)
B1^ms^s	3.6 ± 0.7 2.4–4.8 (*n* = 37)	3.5 ± 0.3 3.0–3.8 (*n* = 7)	5.0 ± 0.5 4.2–5.9 (*n* = 7)	9.2 ± 3.1 6.0–13.0 (*n* = 3)	22.8 ± 2.6 20.0–25.0 (*n* = 2)	1.44 ± 0.17 1.14–1.83 (*n* = 31)	0.33 ± 0.09 0.19–0.56 (*n* = 31)
B1^ml^s	–	–	–	7.0 ± 1.0 6.0–8.0 (*n* = 1)	–	–	–
B2^fs^s	3.4 ± 0.5 2.6–4.4 (*n* = 10)	–	–	10.5 ± 0.5 10.0–11.0 (*n* = 1)	–	–	–
B2^fl^l	–	–	–	13.0 ± 1.5 12.0–14.0 (*n* = 2)	36.0 (*n* = 1)	–	–

**Table 4:** Percentage of successfully hatched pre-larvae (before they started feeding) and completed metamorphosis larvae (calculated by the number of successfully hatched pre-larvae). *n* is the number of crossing experiments.

Type of Crossing	Pre-Larvae		Larvae	
	*n*	Mean ± SD (Range)	*n*	Mean ± SD (Range)
F1^s^	3	31.3 ± 28.7 (10–64)	3	68.3 ± 23.6 (50–95)
F1^l^	1	80.0	1	90.0
F2^s^	12	45.1 ± 33.8 (1–80)	9	70.1 ± 30.9 (1–90)
F2^l^	6	76.6 ± 18.6 (40–90)	5	50.0 ± 23.4 (30–90)
F3^s^	21	2.0 ± 4.3 (0–20)	21	3.0 ± 7.8 (0–30)
F3^l^	11	0.4 ± 0.5 (0–1)	4	0.5 ± 0.6 (0–1)
F4^s^	8	0.4 ± 0.5 (0–1)	3	0
B1^fs^l	3	6.7 ± 5.8 (0–10)	2	6.0 ± 5.7 (2–10)
B1^fs^s	3	27.0 ± 37.5 (1–70)	3	46.7 ± 45.1 (0–90)
B1^fl^s	5	64.2 ± 36.7 (1–90)	5	44.0 ± 28.8.3 (0–80)
B1^fl^l	6	35.1 ± 24.0 (1–70)	6	38.4 ± 36.0 (0–80)
B1^ms^l	6	28.6 ± 21.4 (1–61)	6	53.3 ± 38.3 (10–90)
B1^ms^s	5	53.6 ± 34.6 (1–87)	5	46.7 ± 33.3 (1–90)
B1^ml^l	3	1.7 ± 2.9 (0–5)	1	5.0
B1^ml^s	2	3.0 ± 1.4 (2–4)	–	–
B2^fs^s	4	1.0 ± 2.0 (1–5)	–	–
B2^fl^l	1	5.0	–	–

**Table 5:** Predominant ploidy class (range), fecundity (mean ± SD, range) of females, thickness of egg strings, and egg (=vitellus) diameter (mm) of *Bufotes latastii*, *B. sitibundus*, and their hybrids. *n* is the number of individuals. – no data.

Type of Crossing	Ploidy	Fecundity	Thickness of Egg Strings	Egg Diameter
*latastii*	2 (2)	3069 ± 1829 19–6859 (*n* = 50)	4.4 ± 0.9 2.8–5.8 (*n* = 38)	1.4 ± 0.2 1.0–1.9 (*n* = 381)
*sitibundus*	2 (2)	8583 ± 3451 3814–14,539 (*n* = 25)	3.1 ± 0.7 1.0–4.4 (*n* = 37)	1.4 ± 0.3 0.8–2.3 (*n* = 175)
F1^s^	2 (2–4)	3364 ± 2302 616–7812 (*n* = 18)	4.0 ± 1.0 2.8–6.9 (*n* = 32)	1.7 ± 0.3 1.0–2.8 (*n* = 194)
F1^l^	2 (2–3)	2270 ± 733 854–3753 (*n* = 14)	3.7 ± 0.8 2.6–6.1 (*n* = 65)	1.6 ± 0.3 1.0–2.4 (*n* = 261)
F2^s^	3 (2–4)	2197 ± 1078 739–4162 (*n* = 17)	3.8 ± 0.9 2.1–6.4 (*n* = 85)	1.6 ± 0.4 0.7–2.7 (*n* = 309)
F2^l^	3 (3)	1248 ± 646 567–2523 (*n* = 11)	2.9 ± 0.6 2.0–4.3 (*n* = 62)	1.5 ± 0.4 0.7–2.3 (*n* = 181)
F3^s^	3 (2–3)	2270 ± 1299 703–3775 (*n* = 8)	3.5 ± 0.7 2.0–5.2 (*n* = 38)	1.5 ± 0.3 0.8–2.4 (*n* = 100)
B1^fs^s	3 (2–3)	–	2.5 ± 0.2 2.2–2.9 (*n* = 10)	1.5 ± 0.1 1.2–1.7 (*n* = 20)
B1^fl^l	3 (3)	5790 (*n* = 1)	3.3 ± 0.6 2.8–4.0 (*n* = 5)	2.0 ± 0.1 1.9–2.3 (*n* = 10)

## Data Availability

The original contributions presented in this study are included in the article. Further inquiries can be directed to the corresponding author.
